# New-onset renal diseases after total knee arthroplasty in patients with osteoarthritis: a multicenter retrospective cohort study

**DOI:** 10.7150/ijms.121331

**Published:** 2026-03-17

**Authors:** Shiu-Jau Chen, Hsin-Yo Lu, Chih-Lung Wu, Chun-Wei Lin, Hui-Chin Chang, Shuo-Yan Gau

**Affiliations:** 1 Department of Neurosurgery, MacKay Memorial Hospital, Taipei, Taiwan.; 2 Department of Medicine, MacKay Medical University, New Taipei City, Taiwan.; 3 School of Medicine, Chung Shan Medical University, Taichung, Taiwan.; 4 Department of Orthopedic Surgery, Chung Shan Medical University Hospital, Taichung, Taiwan.; 5 Evidence-based Medicine Center, Chung Shan Medical University Hospital, Taichung, Taiwan.; 6 Library, Chung Shan Medical University Hospital, Taichung, Taiwan.; 7 Department of Medical Education, Ditmanson Medical Foundation Chia-Yi Christian Hospital, Chiayi City, Taiwan.; 8 Department and Graduate Institute of Business Administration, National Taiwan University, Taipei, Taiwan.; 9 Institute of Allergology, Charité-Universitätsmedizin Berlin, Corporate Member of Freie Universität Berlin and Humboldt-Universität zu Berlin, Berlin, Germany.

## Abstract

**Background:**

Osteoarthritis (OA) is a debilitating condition often treated with total knee arthroplasty (TKA), a procedure effective in alleviating pain but associated with potential renal complications, including acute kidney injury (AKI) and chronic kidney disease (CKD). While perioperative risks are known, long-term kidney-related outcomes of TKA remain underexplored. This study investigates the association between TKA and the risks of new-onset AKI and CKD compared to non-surgical OA management.

**Method:**

A retrospective cohort study using the TriNetX Research Network included adults with OA who underwent TKA (n=41,027) and matched non-surgical controls (n=41,027). Propensity score matching balanced demographics, comorbidities, and medical utilization. Renal outcomes were assessed using Hazard ratios (HRs) and 95% confidence intervals (CIs) were evaluated. Sensitivity and stratified analyses evaluated robustness and subgroup-specific risks.

**Results:**

TKA significantly increased the risk of AKI (HR: 1.48, 95% CI: 1.40-1.57) and CKD (HR: 1.30, 95% CI: 1.24-1.37). Females showed higher risks of AKI (HR: 1.55, 95% CI: 1.44-1.67) and CKD (HR: 1.32, 95% CI: 1.24-1.40). Younger patients (18-64 years) exhibited elevated risks of AKI (HR: 1.90, 95% CI: 1.56-2.31) and CKD (HR: 1.73, 95% CI: 1.38-2.18). Sensitivity analyses confirmed consistent results across various models.

**Conclusion:**

TKA is associated with increased risks of AKI and CKD, particularly in females and younger patients. These findings underscore the need for enhanced perioperative care, renal risk stratification, and long-term monitoring to mitigate adverse renal outcomes in TKA recipients.

## Introduction

Osteoarthritis (OA) is a prevalent joint disorder that significantly diminishes individuals' quality of life while placing a notable burden on global healthcare systems 1. This condition primarily affects weight-bearing joints, particularly the knees, and is often characterized by chronic pain and limited mobility 2. For patients with severe knee OA, total knee arthroplasty (TKA) is a widely utilized procedure aimed at alleviating symptoms and enhancing joint function. However, as the demand for TKA grows, concerns about its potential complications have become increasingly prominent 3-5.

Renal complications, including acute kidney injury (AKI) and chronic kidney disease (CKD), are significant risks following major surgeries 6, 7. Factors such as perioperative hypotension, blood loss, nephrotoxic medications, and preexisting health conditions can increase these risks 8. Moreover, OA patients often present with comorbidities like advanced age, obesity, diabetes, and hypertension, which further predispose them to renal outcomes 9. Despite the frequent use of TKA, there remains a gap in understanding the long-term kidney-related risks associated with this surgery compared to non-surgical OA management.

The interplay between OA, TKA, and renal health is complex, with patient characteristics and underlying conditions acting as potential confounders. Some evidence suggests that surgical interventions might help reduce systemic inflammation and related comorbidities 10, yet other studies indicate a possible increased risk of renal complications post-surgery 11. Hence, it is crucial to conduct robust comparative analyses of AKI and CKD risks in patients undergoing TKA versus those managed conservatively. To address these knowledge gaps, we utilize the TriNetX research platform to perform a retrospective cohort analysis. We assessed the incidence of AKI and CKD in OA patients treated with TKA versus those without surgical intervention, while adjusting for variables like age, sex, comorbidities, and medication use.

## Materials and Methods

This retrospective cohort study used a population-based design and drew data from the TriNetX Research Network, a comprehensive global database consolidating electronic medical records from more than 220 healthcare organizations (HCO) in multiple countries 12, 13. Since 2017, the network has expanded substantially from 55 HCOs to more than 220, making it one of the most widely used research platforms globally 14, 15. For this study, we used a subset focused on the US Collaborative Network, which includes records from 69 HCOs within the United States, capturing more than 100 million patient records. The dataset provided detailed clinical information, including demographic data for the patient, diagnoses, medications, and medical procedures. ICD-10-CM codes were employed to identify disease diagnoses, while pharmacological and procedural interventions were classified using Anatomical Therapeutic Chemistry (ATC) and Current Procedural Terminology (CPT) codes. A comprehensive list of the codes used in the study can be found in **Supplementary Table S1**.

The analyses were conducted on patients from January 1, 2005, to December 31, 2023. The case group included patients diagnosed with osteoarthritis who had undergone total knee arthroplasty (TKA). On the contrary, the control group consisted of OA patients who did not have a history of TKA. For non-TKA controls, the index date was defined as the date of a recorded clinical encounter during which a diagnosis of osteoarthritis (ICD-10-CM codes M15-M19) was made. The baseline data presented in** Table 1** were defined as patient characteristics recorded from the beginning of each individual's available history in the database up to three months prior to the index date, and administrative codes as presented in Table S1 are used to define covariates in the baseline. Participants were excluded if they were under 18 years of age or if they had a documented history of cancer or pre-existing AKI, CKD or renal failure, before the index date. The purpose of these exclusions was to decrease the potential biases that were associated with frailty or existing conditions.

The primary exposure of interest in this study was the performance of TKA in patients diagnosed with osteoarthritis. The primary outcome measured was the incidence of renal dysfunction, defined as kidney-related events occurring from three months after the index up to 15 years later. The three-month exclusion window was used to minimize the influence of reverse causality. The methodological choices were reported in detail in **Supplementary Table S2**.

Propensity score matching (PSM) was performed in a 1:1 ratio to ensure that the baseline characteristics between the TKA and the control groups were comparable. The match was performed based on key demographic and clinical factors, including age, sex, race, socioeconomic factors, lifestyle, comorbidities, frequency of emergency department visits, medication usage, healthcare utilization metrics, body mass index (BMI), and relevant laboratory test results. Following PSM, 41,027 individuals were included in each group for analysis. To confirm the robustness of the results, sensitivity analyses were conducted by varying the washout period, follow-up durations, and matched covariate sets. Subgroup analyses were performed to uncover possible risk variations based on age and sex.

Statistical analysis was performed using the TriNetX built-in analytics platform. The risk of renal dysfunction was evaluated using hazard ratios (HR) and corresponding 95% confidence intervals (CI). Standardized mean differences (SMD) were calculated to compare baseline characteristics, with an SMD value greater than 0.1 indicating a notable imbalance. This study complied with the Declaration of Helsinki and the need for informed consent for participants to participate in was waived by the Institutional Review Board of Chung Shan Medical University Hospital (CS1-25004).

## Results

### Baseline Characteristics

**Figure 1:**
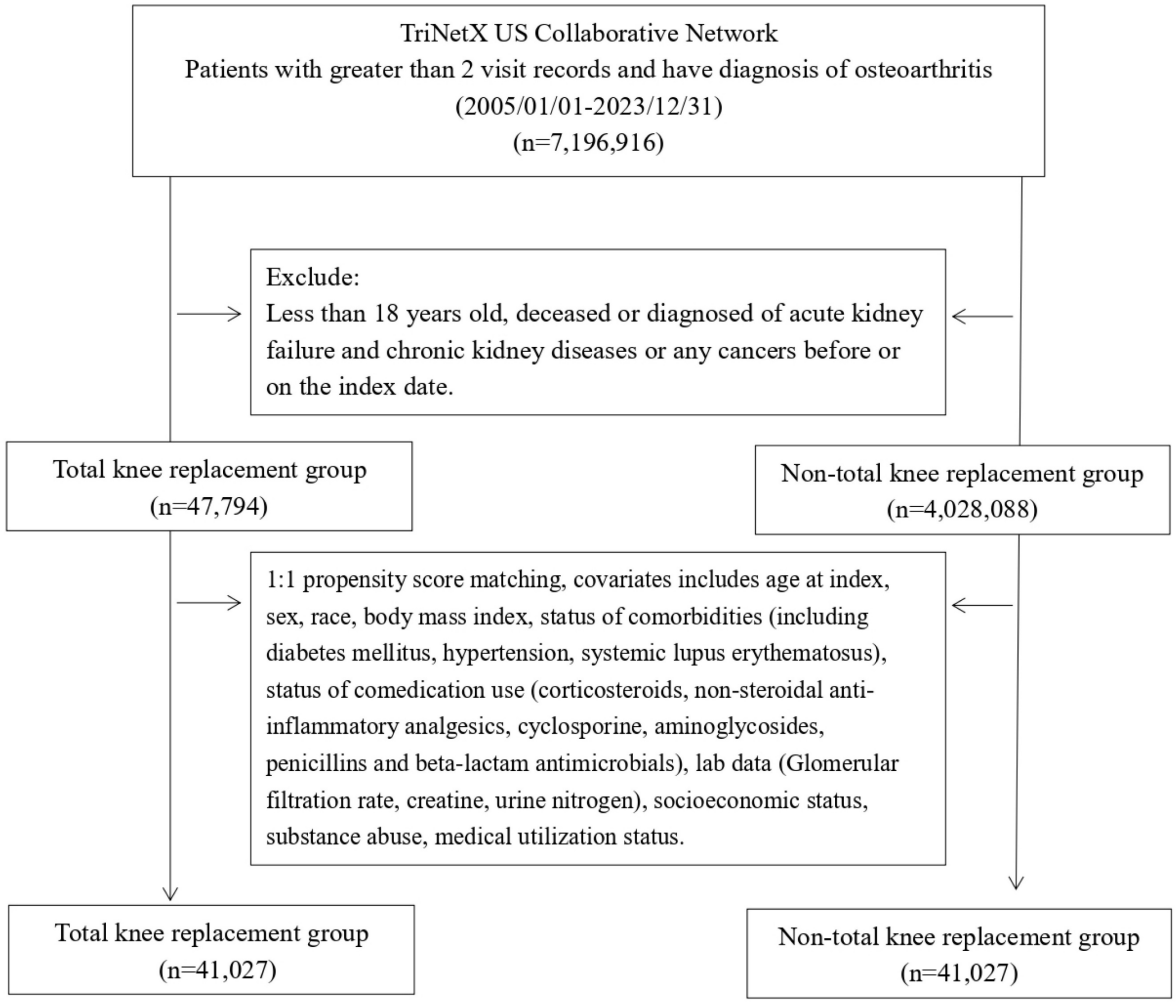
Patient selection process.

Baseline characteristics of the TKA and control cohorts are shown in **Table 1**. After propensity score matching, both cohorts included 41,027 patients (**Figure 1**). Key demographic and clinical features were balanced, including age (mean 61.9 vs. 62.0 years, SMD = 0.01) and sex (60.9 of female in TKA cohort and 61.1% of female in control cohort, SMD = 0.01). The mean follow-up duration remained longer in the TKA cohort compared with the matched control cohort (9.8 ± 4.9 vs. 7.4 ± 4.7 years), Racial distribution and socioeconomic factors showed no significant differences post-matching. Comorbidities and co-medication usage were also well-matched, with SMDs ≤ 0.01 Medical utilization and laboratory data were balanced, ensuring comparability for subsequent analyses.

### New-Onset AKI

**Figure 2:**
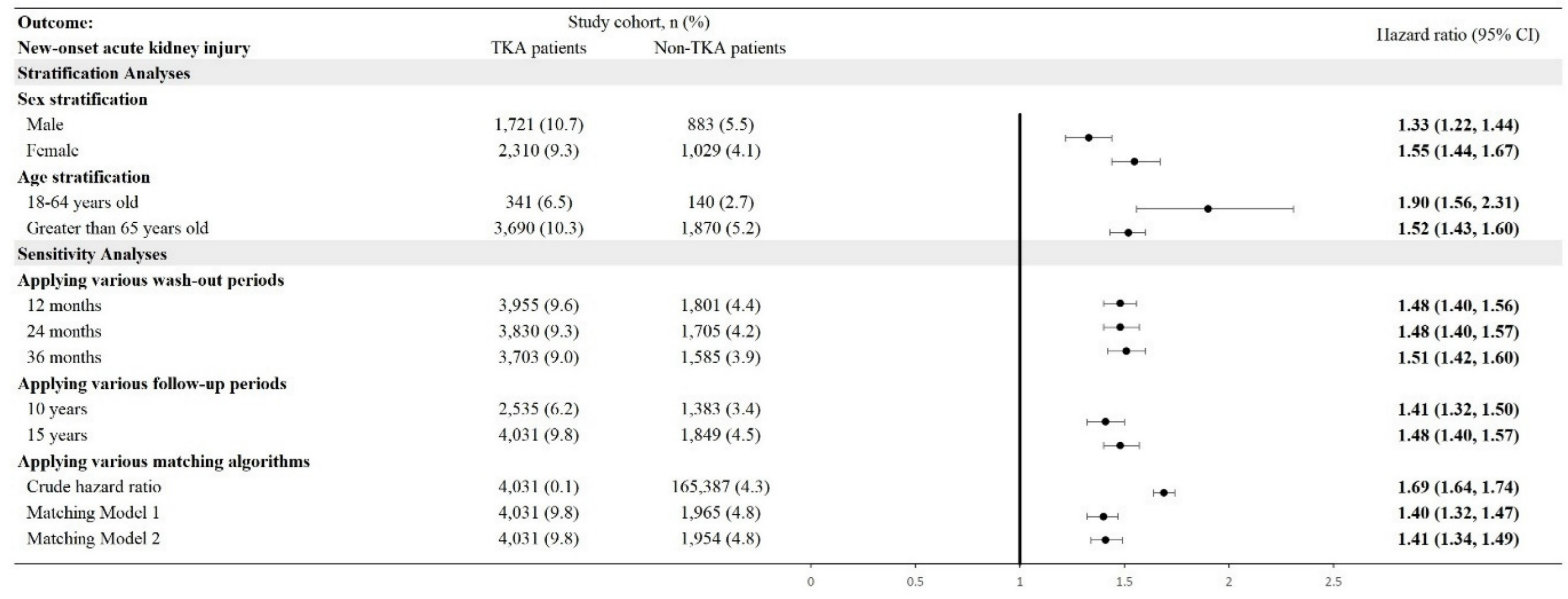
Risk of acute kidney injury in different sensitivity and stratification models. TKA: total knee arthroplasty; 95% CI: 95% confidence interval; Wash-out period: incident outcome event occurred within index date and the specific wash-out period were excluded from analysis; Crude hazard ratio: crude analysis without matching; Matching Model 1: matching covariates including age, sex, race; Matching Model 2: matching covariates including age, sex, race, substance abuse, socioeconomic issues.

**Figure 3:**
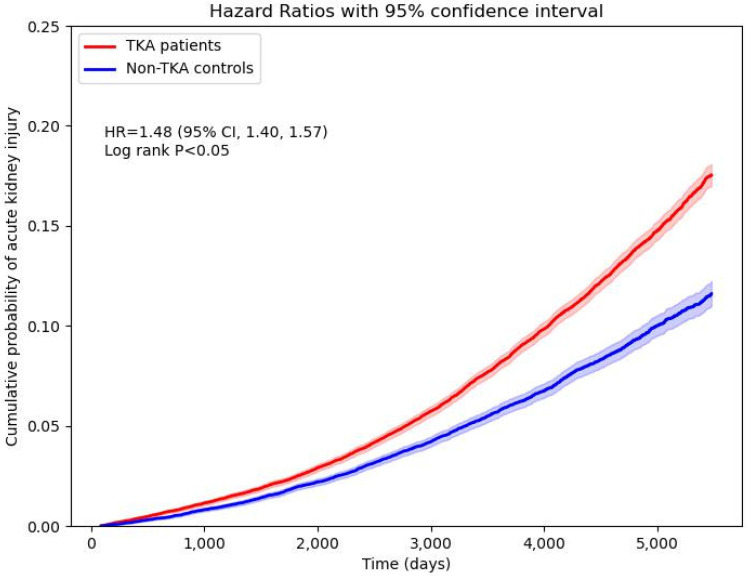
Cumulative probability of new-onset acute kidney injury.

The incidence of new-onset AKI was significantly higher in TKA patients compared to non-TKA patients (**Figure 2**). Stratification by sex revealed an adjusted hazard ratio (HR) of 1.33 (95% CI: 1.22-1.44) for males and 1.55 (95% CI: 1.44-1.67) for females. Age stratification demonstrated the highest risk in patients aged 18-64 years, with an HR of 1.90 (95% CI: 1.56-2.31), compared to an HR of 1.52 (95% CI: 1.43-1.60) in patients older than 65 years. Cumulative probability of AKI increased in the TKA cohort over time, aligning with these stratified hazard ratios (**Figure 3**). Sensitivity analyses consistently supported these findings, with HRs ranging from 1.41 (95% CI: 1.32-1.50) to 1.51 (95% CI: 1.42-1.60) across various wash-out periods (12, 24, and 36 months) and follow-up durations (10 and 15 years).

### New-Onset CKD

**Figure 4:**
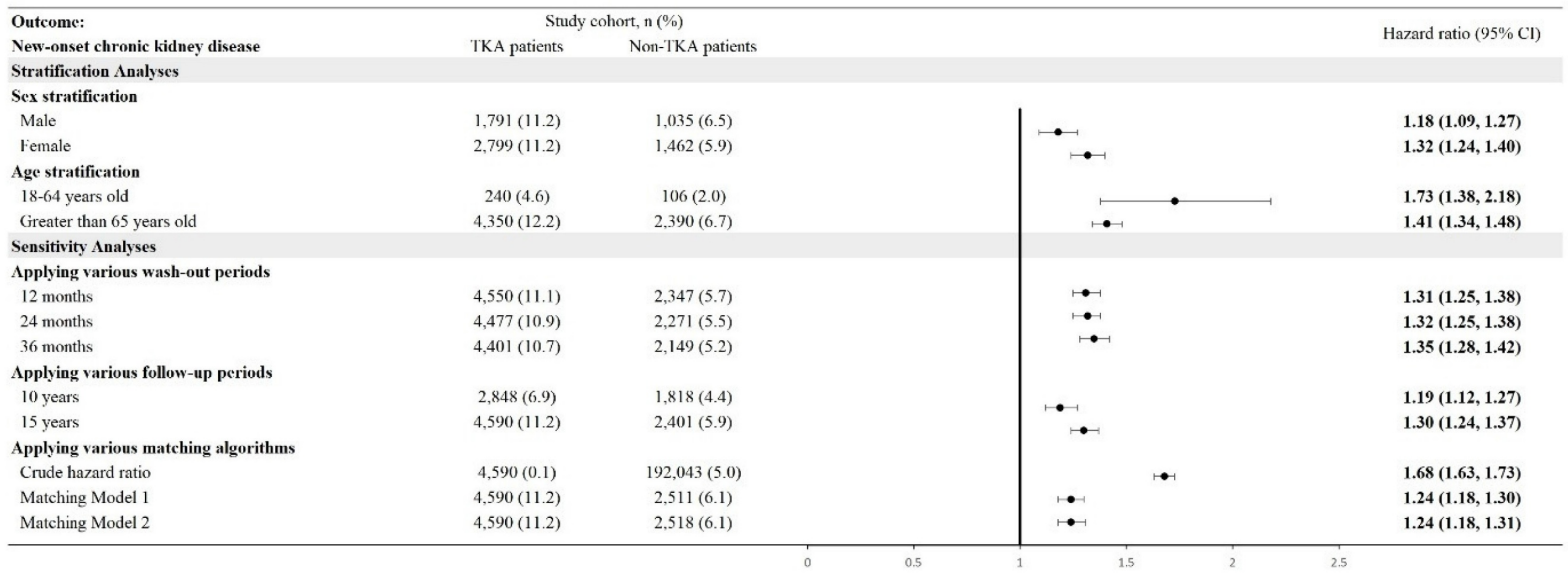
Risk of chronic kidney disease in different sensitivity and stratification models. TKA: total knee arthroplasty; 95% CI: 95% confidence interval; Wash-out period: incident outcome event occurred within index date and the specific wash-out period were excluded from analysis; Crude hazard ratio: crude analysis without matching; Matching Model 1: matching covariates including age, sex, race; Matching Model 2: matching covariates including age, sex, race, substance abuse, socioeconomic issues.

**Figure 5:**
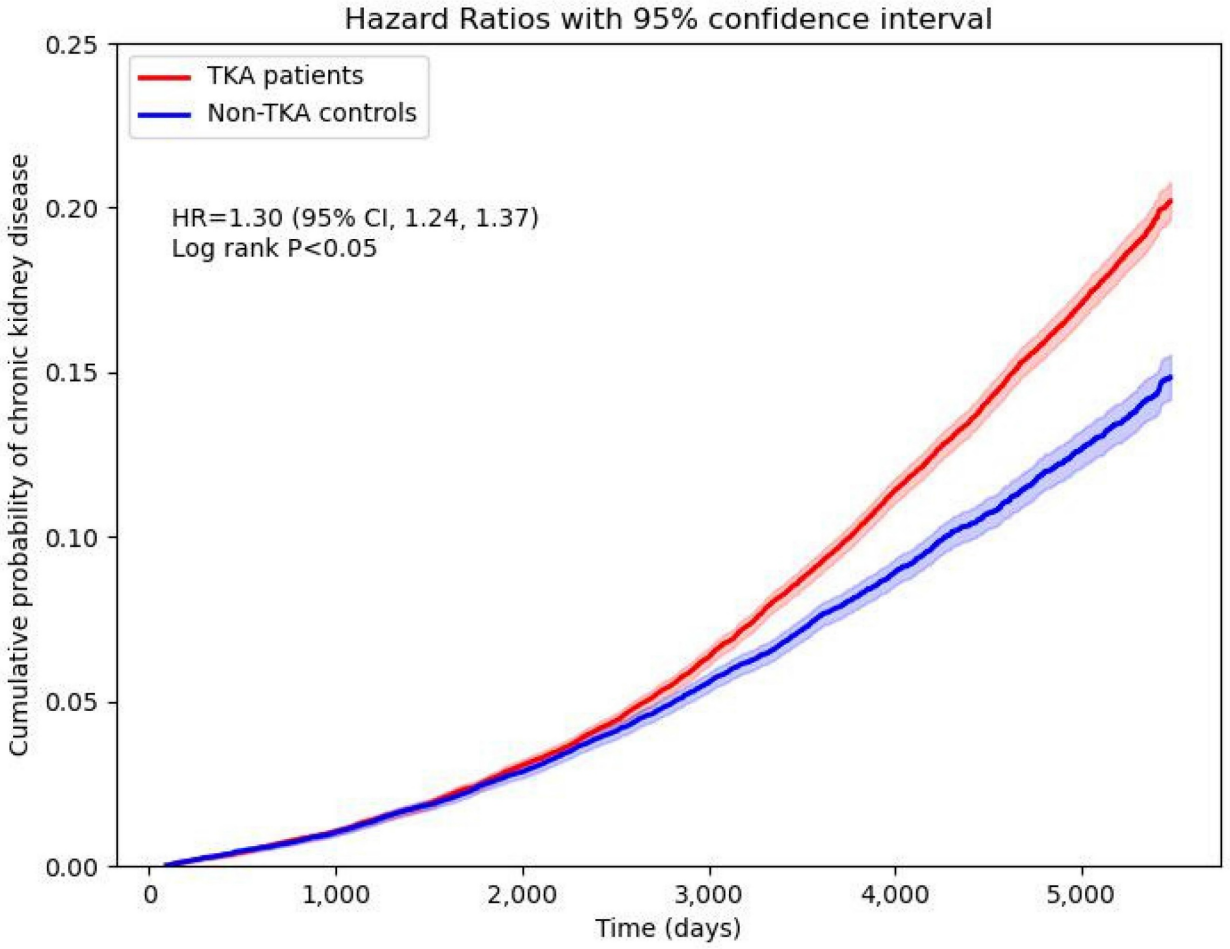
Cumulative probability of new-onset chronic kidney disease.

TKA patients were also at significantly higher risk for new-onset CKD compared to their non-TKA counterparts (**Figure 4**). Stratified analyses revealed an HR of 1.18 (95% CI: 1.09-1.27) for males and 1.32 (95% CI: 1.24-1.40) for females. Age-stratified analyses showed a higher risk in younger patients aged 18-64 years, with an HR of 1.73 (95% CI: 1.38-2.18), while older patients (aged >65 years) exhibited an HR of 1.41 (95% CI: 1.34-1.48). Cumulative probability of CKD also elevated in the TKA cohort over the follow-up period (**Figure 5**). Sensitivity analyses validated these findings, demonstrating HRs ranging from 1.19 (95% CI: 1.12-1.27) to 1.35 (95% CI: 1.28-1.42) across different analytical models, including varying wash-out periods and follow-up times.

## Discussion

This population-based retrospective cohort study demonstrates that patients with osteoarthritis who undergo TKA have a significantly elevated risk of developing AKI and CKD compared to those who do not undergo TKA. After adjustment for baseline characteristics, the risks of new-onset AKI and CKD remained consistent in multiple sensitivity analyzes and follow-up durations, underscoring the robustness of our findings. In particular, our study revealed a higher vulnerability to renal complications among female patients after TKA. These findings highlight the need for increased clinical vigilance and improved perioperative strategies to mitigate renal risks in this population of patients.

TKA is a widely performed surgical procedure that involves the metallic resurfacing of the distal femur and proximal tibia, along with the placement of a polyethylene articulating liner 16. This procedure has become increasingly common in the United States due to its effectiveness in relieving pain and restoring functional quality of life in patients with end-stage osteoarthritis 17. Despite its clinical benefits, TKA is a major surgical intervention associated with significant systemic stress, which can predispose patients to various adverse outcomes, including renal complications 18, 19. Postoperative AKI occurs more frequently in cardiac surgery, where it affects more than a third of patients, while its incidence after orthopedic procedures ranges between 6.7% and 10.8% 20, 21. AKI is among the most common complications associated with major surgeries and often acts as a catalyst for long-term adverse outcomes, such as CKD, end-stage renal disease (ESRD), elevated rates of cardiovascular events, and increased mortality 22-24. AKI does not exist in isolation; instead, it often initiates a cascade of structural and functional changes that lead to a progressive decline of the kidney over time 22.

The increased risk of AKI and CKD after TKA is likely to be due to a combination of surgical stress, the use of perioperative medications, and preexisting comorbidities. TKA-induced surgical stress directly contributes to renal injury through hemodynamic fluctuations, including hypotension and reduced renal perfusion, particularly in cases involving significant blood loss, prolonged operative time, or tourniquet application 25, 26. These events can result in an ischemia-reperfusion injury that causes acute tubular necrosis (ATN), the predominant pathophysiological mechanism of AKI 23, 26. Furthermore, systemic inflammation and oxidative stress triggered by surgical trauma exacerbate renal injury and increase the risk of AKI and subsequent progression of CKD 27.

In addition to surgical factors, the perioperative use of nephrotoxic medications plays a central role in increasing renal risks. NSAIDs, widely prescribed for their analgesic and anti-inflammatory properties, inhibit prostaglandin synthesis, thus reducing renal blood flow and increasing the risk of AKI 23, 28. Prolonged or excessive use can further accelerate the progression of CKD. Similarly, nephrotoxic antibiotics and anesthetics administered during the perioperative period can contribute to renal injury, particularly in patients with underlying renal vulnerability 21.

Patients who undergo TKA are also more likely to have multiple preexisting comorbidities, such as diabetes mellitus, hypertension, and obesity, which are established risk factors for renal dysfunction 25, 29. The combination of surgical stress and preexisting systemic conditions makes the kidneys more susceptible to injury. Furthermore, elderly patients, who make up a significant proportion of TKA recipients, are particularly vulnerable due to age-related decreases in renal reserve and reduced recovery capacity 16. For these individuals, even transient episodes of AKI can result in incomplete renal recovery, progressing to visible CKD or ESRD over time.

Previous studies have identified the gender of the male as a preoperative risk factor for PO-AKI 21 ; however, our sensitivity analysis revealed that female patients are particularly at risk for renal complications after TKA. Although the underlying mechanisms are unclear, several factors may contribute to this observation. Postmenopausal women experience a decrease in estrogen levels, a hormone known to protect kidney function by maintaining mitochondrial homeostasis and regulating the endothelin-1 (ET-1) system within the kidneys 30. The loss of this hormonal protection can make female patients more susceptible to renal injury 31. Furthermore, women have smaller kidney volumes compared to men, which can exacerbate their vulnerability to renal stress during periods of hemodynamic instability 32. Differences in drug metabolism may also play a role, as women may exhibit an increased sensitivity to nephrotoxic medications, such as NSAIDs and antibiotics, commonly used in the perioperative setting 28, 33. However, more large-scale prospective studies are warranted to elucidate the mechanisms underlying this gender-based disparity.

Although older people represent a high-risk group for AKI and CKD due to age-related decline in renal function and the presence of multiple comorbidities, it is important to recognize that younger patients (18 to 64 years) are not immune to renal complications 16. In younger populations, predisposing factors such as trauma-related TKA, the use of nephrotoxic drugs, and unrecognized preexisting renal abnormalities can contribute to the risk of AKI. Lifestyle factors, such as smoking, alcohol consumption, and substance use, can further exacerbate renal injury 34, 35. These findings suggest that younger and older patients with TKA need comprehensive perioperative evaluation and follow-up to minimize renal risks.

Our results align with previous studies reporting increased renal complications after major surgical interventions. Although TKA remains an effective and necessary treatment to improve quality of life in patients with OA, our findings emphasize the need to carefully balance the benefits of surgery against the potential risks of kidney injury. For patients with pre-existing risk factors, such as diabetes, hypertension, and advanced age, perioperative strategies should focus on minimizing renal stress and improving recovery.

Clinically, these findings have important implications for the treatment of patients with TKA patients 18, 24, 25. Comprehensive preoperative evaluation of renal function is essential to identify high-risk individuals and optimize preexisting comorbidities. Strategies such as meticulous intraoperative hemodynamic management and fluid optimization can reduce the risk of renal ischemia. Minimally invasive surgical techniques and optimized operating time may further mitigate surgical stress. Postoperatively, prudent use of NSAIDs and other nephrotoxic medications is paramount, particularly for patients with baseline renal failure. Alternative analgesics must be considered to minimize renal burden. Furthermore, early and close monitoring of renal function using serum creatinine, eGFR, and urine output can facilitate timely identification of AKI and prompt intervention, preventing further deterioration.

This study has several strengths, including its large sample size, rigorous matching of propensity scores to reduce confounding bias, and long-term follow-up, which collectively improve the reliability of our findings. However, several limitations should be acknowledged. First, as a retrospective study based on electronic health records, the possibility of information bias and misclassification bias cannot be eliminated. Renal outcomes in this study were defined using ICD-10-CM codes, which may introduce misclassification bias. Although the TriNetX platform includes certain laboratory values such as serum creatinine, eGFR and albumin in urine, only single measurements are available for outcome definition, and repeated assessments required for robust CKD diagnosis are not feasible. Consequently, outcome ascertainment could not be based on laboratory measures, and this limitation should be considered when interpreting our findings. Second, the study lacks granular data on perioperative variables, such as fluid management, blood loss, and medication dosages. The potential for residual confounding due to unmeasured residual confounders should be prudently considered, as these unmeasured factors may directly affect renal outcomes and could therefore exert a substantial influence on our results. In future studies, information on these data is also critical to understanding the mechanisms underlying association between TKA and renal dysfunction. Confounding by disease severity should also be considered. Patients who underwent TKA likely represented individuals with more advanced OA severity or greater functional impairment. These characteristics may themselves be associated with renal outcomes and could therefore confound the observed associations. However, the TriNetX platform does not provide information on clinical grading or severity indices of OA, preventing us from adjusting for this factor. In addition, our analyses were restricted to baseline covariates and did not account for time-varying confounders. The database does not allow dynamic assessment of new comorbidities or cumulative exposure to nephrotoxic medications after TKA. These limitations should be considered when interpreting the results. Third, the predominance of white participants in the cohort limits the generalizability of our findings to other racial and ethnic groups. Fourth, due to the retrospective cohort nature of the current study, we are not able to confirm causality. While our comparison was between TKA patients and non-surgical OA patients, it is possible that the observed increased risk of kidney injury is related to general surgical stress rather than TKA-specific mechanisms. Fifth, in our analysis, patients with documented death events were excluded from the study cohort to avoid ambiguity in outcome ascertainment. As a result, competing risk models accounting for mortality could not be applied. Sixth, our analyses primarily relied on time-averaged hazard ratios and single cumulative incidence curves, which assume proportional hazards. This approach may not fully reflect how the risk of CKD evolves over time following TKA. Future research applying time-varying effect models would be valuable to clarify the temporal dynamics of this association. Seventh, our study compared TKA recipients with non-surgical OA patients. As such, the observed associations may reflect the general burden of major surgery rather than mechanisms unique to TKA. Eighth, we acknowledge that the mean follow-up duration was longer in the TKA cohort than in the non-surgical OA cohort, both before and after propensity score matching. Differences in follow-up time may introduce potential bias, as longer observation periods inherently increase the likelihood of detecting outcome events, which could lead to an overestimation of renal risk in the cohort with extended follow-up. Several factors may contribute to this imbalance. Patients undergoing total knee arthroplasty often remain under long-term postoperative surveillance and have sustained engagement with healthcare systems, whereas non-surgical osteoarthritis patients may have more intermittent clinical encounters. Such differences in healthcare utilization patterns can influence longitudinal data availability in real-world electronic health record-based studies and result in unequal follow-up durations between comparison groups. Although sensitivity analyses using predefined follow-up windows demonstrated consistent associations, residual bias related to differential follow-up time cannot be completely excluded. This limitation should be considered when interpreting the magnitude of the observed associations between TKA and subsequent risks of acute kidney injury and chronic kidney disease.

In conclusion, this study highlights a significant association between TKA and the increased risk of AKI and CKD, particularly in female patients. Although TKA remains a critical intervention for the management of OA, these findings emphasize the importance of proactive perioperative care to minimize renal complications. A multidisciplinary approach that involves preoperative risk assessment, intraoperative hemodynamic optimization, and vigilant postoperative monitoring is essential to ensure patient safety. Future research should focus on identifying new biomarkers for early detection of AKI, evaluating alternative pain management strategies, and exploring targeted interventions to protect renal function in high-risk patients with TKA. By addressing these challenges, clinicians can maximize the benefits of TKA while minimizing its long-term renal impact.

## Supplementary Material

Supplementary tables.

## Figures and Tables

**Table 1:** Baseline characteristics

	Before matching				After matching^a^			
	TKA cohort (n=47,794)	Control cohort (n=4,028,088)	SMD	p-value	TKA cohort (n=41,027)	Control cohort (n=41,027)	SMD	p-value
Mean follow-up years ±SD	9.8±4.9	7.7±4.8	0.44	0.00	9.8±4.9	7.4±4.7	0.50	0.00
**Age at index**								
Mean±SD	61.9±10.6	57.0±15.1	0.38	0.00	61.9±10.6	62.0±10.8	0.00	0.77
**Sex**								
Male	16044 (39.1)	1546568 (40.6)	0.03	0.00	16044 (39.1)	15944 (38.9)	0.00	0.47
Female	24966 (60.9)	2257655 (59.3)	0.03	0.00	24966 (60.9)	25063 (61.1)	0.00	0.49
Unknown Gender	17 (<0.1)	2165 (0.1)	0.01	0.19	17 (<0.1)	20 (<0.1)	0.00	0.62
**Race, n (%)**								
White	29795 (72.6)	2801033 (73.6)	0.02	0.00	29795 (72.6)	29830 (72.7)	0.00	0.78
Black or African American	5565 (13.6)	535428 (14.1)	0.01	0.00	5565 (13.6)	5697 (13.9)	0.01	0.18
Asian	1846 (4.5)	96176 (2.5)	0.11	0.00	1846 (4.5)	1289 (3.1)	0.07	0.00
American Indian or Alaska Native	264 (0.6)	19848 (0.5)	0.02	0.00	264 (0.6)	187 (0.5)	0.03	0.00
Native Hawaiian or Other Pacific Islander	494 (1.2)	14988 (0.4)	0.09	0.00	494 (1.2)	166 (0.4)	0.09	0.00
Other Race	828 (2.0)	113567 (3.0)	0.06	0.00	828 (2.0)	1210 (2.9)	0.06	0.00
Unknown Race	2235 (5.4)	225348 (5.9)	0.02	0.00	2235 (5.4)	2648 (6.5)	0.04	0.00
**Socioeconomic status**								
Problems related to education and literacy	0 (<0.1)	180 (<0.1)	0.01	0.16	0 (<0.1)	0 (<0.1)		
Problems related to employment and unemployment	10 (<0.1)	770 (<0.1)	0.00	0.56	10 (<0.1)	10 (<0.1)	0.00	1.00
Occupational exposure to risk factors	0 (<0.1)	447 (<0.1)	0.02	0.03	0 (<0.1)	10 (<0.1)	0.02	0.00
Problems related to housing and economic circumstances	10 (<0.1)	2632 (0.1)	0.02	0.00	10 (<0.1)	10 (<0.1)	0.00	1.00
**Lifestyle**								
Mental and behavioral disorders due to psychoactive substance use	725 (1.8)	111548 (2.9)	0.08	0.00	725 (1.8)	736 (1.8)	0.00	0.77
**Comorbidities**								
Hypertension	5992 (14.6)	411054 (10.8)	0.11	0.00	5992 (14.6)	5947 (14.5)	0.00	0.66
Diabetes mellitus	1732 (4.2)	150477 (4.0)	0.01	0.01	1732 (4.2)	1741 (4.2)	0.00	0.88
Systemic lupus erythematosus	55 (0.1)	6128 (0.2)	0.01	0.18	55 (0.1)	41 (0.1)	0.01	0.15
**Co-medications**								
Corticosteroids	1336 (3.3)	146644 (3.9)	0.03	0.00	1336 (3.3)	1268 (3.1)	0.01	0.18
Non-steroidal anti-inflammatory analgesics	743 (1.8)	89034 (2.3)	0.04	0.00	743 (1.8)	747 (1.8)	0.00	0.92
Cyclosporine	32 (0.1)	3094 (0.1)	0.00	0.82	32 (0.1)	29 (0.1)	0.00	0.70
Aminoglycosides	285 (0.7)	13568 (0.4)	0.05	0.00	285 (0.7)	258 (0.6)	0.01	0.25
Penicillins and beta-lactam antimicrobials	2317 (5.6)	112960 (3.0)	0.13	0.00	2317 (5.6)	2311 (5.6)	0.00	0.93
**Medical Utilization Status**								
Ambulatory visit	20684 (50.4)	2095249 (55.0)	0.09	0.00	20684 (50.4)	20611 (50.2)	0.00	0.61
Emergency Department Services	2043 (5.0)	337049 (8.9)	0.15	0.00	2043 (5.0)	2093 (5.1)	0.01	0.42
Inpatient Encounter	2335 (5.7)	243307 (6.4)	0.03	0.00	2335 (5.7)	2272 (5.5)	0.01	0.34
**Laboratory data**								
BMI, n (%)								
≧ 25 (kg/m^2^)	7506 (18.3)	683891 (18.0)	0.01	0.08	7506 (18.3)	7465 (18.2)	0.00	0.71
Creatine, n (%)					10 (<0.1)	10 (<0.1)		
≧ 1.5 (mg/dl)	10 (<0.1)	15 (<0.1)	0.00	0.69	0 (<0.1)	10 (<0.1)	0.02	0.00
Urine nitrogen, n (%)					11 (<0.1)	10 (<0.1)		
≧ 25 (mg/dl)	10 (<0.1)	336 (<0.1)	0.01	0.00	10 (<0.1)	10 (<0.1)	0.00	1.00
Glomerular filtration rate, n (%)					45 (0.1)	172 (0.4)		
0 to 60 (mL/min/1.73m^2^)	29 (0.1)	4222 (0.1)	0.01	0.01	29 (0.1)	26 (0.1)	0.00	0.69

TKA: total knee arthroplasty; SD: standardized deviation; SMD: standardized mean difference In TriNetX system, if population is less or equal than 10, the data will be presented as 10 for privacy protection. ^a^ 1:1 propensity score matching, covariates includes age at index, sex, race, body mass index, status of comorbidities (including diabetes mellitus, hypertension, systemic lupus erythematosus), status of comedication use (corticosteroids, non-steroidal anti-inflammatory analgesics, cyclosporine, aminoglycosides, penicillins and beta-lactam antimicrobials), lab data (Glomerular filtration rate, creatine, urine nitrogen), socioeconomic status (problems related to education and literacy, problems related to employment and unemployment, occupational exposure to risk factors, problems related to housing and economic circumstances), substance abuse (mental and behavioral disorders due to psychoactive substance use), medical utilization status (ambulatory visit, emergency department service, inpatient encounter).
