# Supplementary material for: New-onset renal diseases after total knee arthroplasty in patients with osteoarthritis: a multicenter retrospective cohort study
Source: Int J Med Sci. 2026 Mar 17;23(4):1509–18. doi: 10.7150/ijms.121331 (PMC13048896; doi:10.7150/ijms.121331)
Supplement: Supplementary file 1 — Supplementary tables. [file ijmsv23p1509s1.pdf]

Supplementary Files

Supplementary Tables

Table S1. Utilized definitions of study population, covariates and outcome events

**Description**	**Adopted codes** [a]
Study Population	
Osteoarthritis	ICD-10-CM: M15-M19
Procedures	
Total knee replacement	ICD-10-PCS[b] : 0SRD0JZ, 0SRC0JZ, 0SRW0JZ, 0SRV0JZ, 0SRU0JZ, 0SRT0JZ, 0SRD07Z, 0SRD0KZ, 0SRU07Z, 0SRU0KZ, 0SRW07Z, 0SRW0KZ, 0SRC0KZ, 0SRT07Z, 0SRT0KZ, 0SRV0KZ
Confounding factors and comorbidities	
Neoplasms	ICD-10-CM: C00-D49
Diabetes mellitus	ICD-10-CM: E08-E13
Hypertension	ICD-10-CM: I10
Systemic lupus erythematosus	ICD-10-CM: M32
Mental and behavioral disorders due to psychoactive substance use	ICD-10-CM: F10-F19
Problems related to education and literacy	ICD-10-CM: Z55
Problems related to employment and unemployment	ICD-10-CM: Z56
Occupational exposure to risk factors	ICD-10-CM: Z57
Problems related to housing and economic circumstances	ICD-10-CM: Z59
Co-Medications	
Corticosteroids	ATC code: R01AD

Non-steroidal anti-inflammatory analgesics	VA code: CN104
Cyclosporine	RxNorm:3008
Aminoglycosides	VA code:AM300
Penicillins and beta-lactam antimicrobials	VA code: AM114
Outcomes	
Acute kidney injury	ICD-10-CM: N17
Chronic kidney disease	ICD-10-CM: N18

[a]ICD-10-CM: International Classification of Diseases, Tenth Revision, Clinical Modification

[b]ICD-10-PCS: International Classification of Diseases, Tenth Revision, Procedure Coding System

Table S2. Description of applied sensitivity analysis models

Models	Description	Corresponding figures
**Applying different follow-up time after index date**		
10 years /15 years after index date	Only events related to outcomes that occur during the established follow-up timeframe will be considered in the analysis. Additionally, each sub-analysis included a consistent three-month washout period.	**Figures 2 and 4**
**Applying different wash-out period after index date**		
12 months/24 months/36 months after index date	Outcome events that arise within the specified washout timeframe will not be considered in the analysis. Furthermore, a uniform follow-up span of 15 years was applied to each sub-analysis.	**Figures 2 and 4**
**Applying different matching covariates**		
Crude model	The analysis was conducted without the use of propensity score matching.	**Figures 2 and 4**
Matching model 1	The variables considered for matching were age at the time of index, sex, and racial background.	
Matching model 2	The variables considered for matching were age at the time of index, sex, racial background, substance abuse, socioeconomic issues	
